# A generalised catalytic model to assess changes in risk for multiple reinfections with SARS-CoV-2

**DOI:** 10.1371/journal.pone.0315476

**Published:** 2025-01-02

**Authors:** Belinda Lombard, Cheryl Cohen, Anne von Gottberg, Jonathan Dushoff, Juliet R. C. Pulliam, Cari van Schalkwyk

**Affiliations:** 1 South African DSI-NRF Centre of Excellence in Epidemiological Modelling and Analysis (SACEMA), Stellenbosch University, Stellenbosch, South Africa; 2 Centre for Respiratory Diseases and Meningitis, National Institute for Communicable Diseases, a division of the National Health Laboratory Health Laboratory Service, Johannesburg, South Africa; 3 School of Public Health, Faculty of Health Sciences, University of the Witwatersrand, Johannesburg, South Africa; 4 School of Pathology, Faculty of Health Sciences, University of the Witwatersrand, Johannesburg, South Africa; 5 Department of Biology, McMaster University, Hamilton, Ontario, Canada; Texas A&M University College Station, UNITED STATES OF AMERICA

## Abstract

**Background:**

Monitoring trends in multiple infections with SARS-CoV-2, following several pandemic waves, provides insight into the biological characteristics of new variants, but also necessitates methods to understand the risk of multiple reinfections.

**Objectives:**

We generalised a catalytic model designed to detect increases in the risk of SARS-CoV-2 reinfection, to assess the population-level risk of multiple reinfections.

**Methods:**

The catalytic model assumes the risk of reinfection is proportional to observed infections and uses a Bayesian approach to fit model parameters to the number of *n*^*th*^ infections among individuals that occur at least 90 days after a previous infection. Using a posterior draw from the fitted model parameters, a 95% projection interval of daily *n*^*th*^ infections is calculated under the assumption of a constant *n*^*th*^ infection hazard coefficient. An additional model parameter was incorporated for the increased reinfection risk detected during the Omicron wave. The generalised model’s performance was then assessed using simulation-based validation.

**Key findings:**

No additional increase in the risk of third infection was detected after the increase detected during the Omicron wave. Using simulation-based validation, we show that the model can successfully detect increases in the risk of third infections under different scenarios.

**Limitations:**

Even though the generalised model is intended to detect the risk of *n*^*th*^ infections, it is validated specifically for third infections, with its applicability for four or more infections being unconfirmed. Furthermore, the method’s sensitivity to low counts of *n*^*th*^ infections, limits application in settings with small epidemics, limited testing coverage or early in an outbreak.

**Conclusions:**

The catalytic model was successfully adapted to detect increases in the risk of *n*^*th*^ infections, enhancing our capacity to identify future changes in the risk of *n*^*th*^ infections by SARS-CoV-2 or other similar pathogens.

## Introduction

In March 2020, the World Health Organization declared a global pandemic of COVID-19 caused by severe acute respiratory syndrome coronavirus 2 (SARS-CoV-2) [1], prompting extensive mathematical modelling efforts to understand transmission dynamics and assess the global spread of the disease [2]. Understanding the risk of multiple reinfections is crucial, in light of waning immunity and the emergence of new variants, which could enhance SARS-CoV-2 spread through previously infected individuals.

Prior research has laid the foundation for understanding SARS-CoV-2 reinfection dynamics. Wangari *et al*. examined reinfection transmission mechanisms using a compartmental model [3]. Another study developed a model to validate a test-negative study design, which revealed that protection against reinfection was higher when the primary infection was caused by the Alpha variant compared to the Beta variant [4].

Pulliam *et al*.’s catalytic model [5] represents another pivotal development. Using line list data of all observed infections in South Africa, the model assumed that the reinfection hazard was proportional to the seven-day moving average of detected cases, with a constant hazard coefficient. By comparing the projected with observed reinfections during the projection period, it assessed potential changes in the reinfection hazard coefficient. The study identified an increase in reinfection hazard during the Omicron wave in November 2021, providing the first epidemiological evidence of the Omicron variant’s increased reinfection risk compared to previous variants [5].

The noticeable increase in reinfections during South Africa’s Omicron wave prompts the investigation of the risk of multiple reinfections (three or more infections). Our study generalises the model developed by Pulliam *et al*. to detect increases in the risk of multiple reinfections in South Africa. The original model findings, complemented by the findings for third infections from the extended model presented here, have been applied to South African data and published in the National Institute for Communicable Diseases (NICD) monthly report on SARS-CoV-2 Reinfection Trends in South Africa [6].

Our study contributes to the understand of practical immune dynamics of SARS-CoV-2, potentially informing vaccination policies [7] and the identification of emerging immune-escape variants of SARS-CoV-2.

## Methodology

### Data source

The dataset used in this study is a time series of the daily counts of primary infections, second infections, third infections and fourth infections of SARS-CoV-2 in South Africa from 4 March 2020 to 29 November 2022. This dataset, as detailed in Pulliam *et al*. [5] is accessible on Zenodo (DOI: 10.5281/zenodo.7426515).

The observed infections in the dataset [5] were obtained from a national dataset containing all positive tests in South Africa, detected by either polymerase chain reaction (PCR) or rapid antigen tests (RATs). Reporting of positive tests by laboratories was mandatory, although RATs are known to have been underreported. In the dataset, deterministic and probabilistic linkage methods were used to identify repeated tests of the same person. Positive tests of an individual that were at least 90 days after the most recent positive test from the previously observed infection were assumed to represent new infections. This delay is introduced to distinguish reinfection from prolonged viral shedding [8, 9]. The specimen receipt date was used as the date of reference in the analysis. In this study, we focus on the number of third infections (*n* = 3).

### The generalised model

The adapted model calculates the number of expected *n*^*th*^ infections on day *x* from prior (*n*−1)^*th*^ infections reported at least 90 days prior to day *x* without subsequent detection.

Building on the original catalytic model, with *t* the last date of testing positive for the (*n*−1)^*th*^ infection, the cumulative hazard through day *x* of an individual is calculated as:

$$H(t,x)=\lambda_{n-1}\sum_{i=t+90}^{i=x}{Î}_{i}^{tot}$$

where ${Î}_{i}^{tot}$ is the 7-day moving average of total infections reported on day *i* and *λ*_*n*−1_ is a *n*^*th*^ infection fitted coefficient describing hazard experienced by individuals with *n*−1 prior infections.

From this, the probability of an *n*^*th*^ infection by day *x*, given a previous (*n*−1)^*th*^ reported infection on day *t* is described as:

$$p_{n}(t,x)={1-e}^{-H(t,x)}$$

The expected number of *n*^*th*^ infections, *Y*_*n*,*x*_ reported *by* day *x* is calculated by summing over possible dates for the (*n*−1)^*th*^ infection, calculated as:

$$Y_{n,x}=\sum_{t=0}^{t=x}I_{n-1,t}p_{n}(t,x)$$

where *I*_*n*−1,*t*_ is the number of (*n*−1)^*th*^ infections reported on day *t*. The expected number of *n*^*th*^ infections *on* day *x* can then be calculated as:

$$D_{x}=Y_{n,x}-Y_{n,x-1}.$$

We used this model to assess third infection risk in South Africa from March 2020 to November 2022 and performed simulation-based validation under a broad range of scenarios to evaluate the performance of the method.

### Fitting the model to South African data on third infections (*n* = 3)

The model assumes that the number of reinfections follows a negative binomial distribution with a mean denoted by *D*_*x*_. In Pulliam et al., two parameters—the hazard coefficient (*λ*_1_) and the inverse of the negative binomial dispersion parameter (*κ*_1_)—were fitted to the data up to 28 February 2021. These fits were projected forward and by comparing projections to observations an increase in the risk of a second infection was detected during the Omicron wave (after 31 October 2021). When considering the risk of third infections (*n* = 3), the generalised model’s parameters did not converge over the same fitting period due to low observation of third infections before the Omicron wave (S1 Fig in S1 File). To overcome this issue, the fitting period was extended to 31 January 2022 and an additional parameter ${(\lambda }_{2}^{\prime })$ was introduced to account for the increased risk of a second infection with the Omicron variant [5].

The probability of having a third infection reported by day *x*, given that the person had a positive test for a second infection on day *t*, can then be calculated as:

$$p_{2}(t,x)=1-e^{-\lambda_{t,2}\sum_{i=t+90}^{i=x}{Î}_{i}^{\prime }}$$

where

$\lambda_{t,2}=\{\lambda_{2}\;if\;i\leq t_{1};\;{\lambda^{\prime }}_{2}\;if\;i>t_{1}\}$ and *t*_1_ = 31 October 2021.

### Model fitting and projection

Model parameters were fitted using Monte Carlo Markov Chains (MCMC). We used 10,000 iterations and four chains, discarding the first 1,500 iterations as burn-in for each chain. We specified uninformative prior distributions over the ranges 1.2∙10^−9^ to 1.75∙10^−7^ for *λ*_*n*−1_ and $\lambda_{n-1}^{\prime }$, and 0.001 to 2 for *κ*_*n*−1_ (the selected values are similar to the ranges chosen in [5]). Convergence of the parameters was measured using Gelman-Rubin diagnostics with the `gelman.diag`function from the *coda* package in R [10]. Gelman-Rubin compares the within-chain and between-chain variance to evaluate the Monte Carlo Markov Chains, as this gives an indication of whether the initial value has been “forgotten”. A value of less than 1.1 indicates a low difference between the variances and, therefore, convergence [11, 12].

The projected *n*^*th*^ infections were calculated from a joint posterior distribution from the chains that were fitted during the MCMC procedure, with 2,000 equally spaced samples drawn from the four chains (after discarding burn-in). For each model parameter combination from the posterior distribution, 100 stochastic simulations were run to calculate the number of expected third infections for each day up until 29 November 2022 for that parameter combination. From the realisations, two projection intervals are calculated: the middle 95% of the expected daily *n*^*th*^ infections, and the middle 95% of the 7-day moving average of expected *n*^*th*^ infections.

### Model validation

In [13], we conducted simulation-based validation to assess the performance of the original catalytic model when introducing changes in the risk of second infections under different scenarios.

We concluded that the model is robust to several important aspects of the observation process that are not directly accounted for in the model. Here, we assessed the model by performing sensitivity analyses on the model’s suitability for assessing third infection risk under different increases in the risk of second and third infections.

To validate the *n*^*th*^ infection method proposed in this study, we considered a simulated dataset of primary infections. The calculation of the simulated dataset was extended from [13] where the seven-day moving average of infections from South African data (available from [14]) was increased by a factor of 5 and subjected to negative binomial sampling with a shape parameter of 1/*κ*, where *κ*≈0.27 was the median of the posterior sample in Pulliam et al. Since the method was shown to be robust for different observation probabilities for primary infections and reinfections [13], we considered fixed primary infection, second infection, and third infection observation probabilities (0.2, 0.5 and 0.35 respectively) for this analysis. We tested the performance of the model on simulated data in different data-generation scenarios by varying the difference in reinfection risk (both the second infection and third infection risk) between a pre-Omicron-like period and a later Omicron-like period. This approach determined whether the model could accurately 1) fit the model parameters for third infections ($\lambda_{2},\;\lambda_{2}^{\prime }$ and *κ*_2_), and 2) detect simulated changes in the risk of third infection.

Similar to [13], we generated a timeseries for the number of observed second infections and third infections from the simulated timeseries of primary infections, by drawing a binomial random variable based on the observation probabilities. Using the observed *k*^*th*^ infections, the number of (*k*+1)^*th*^ infections was calculated using a unique hazard coefficient for each *k*>1. In this simulation-based validation, we calculated a time series for observed primary infections (*k* = 1), observed second infections (*k* = 2) and observed third infections (*k* = 3). More information on how the simulated dataset was derived is available in the supplementary material.

The hazard coefficients for second and third infections were modified using two scale parameters to represent different increases in the reinfection risk over time: the first increase, introduced by scale parameter *σ*_1_, was introduced to represent the Omicron wave (after 31 October 2021) with no subsequent increase in reinfection risk (*σ*_1_ varied between 1 and 3; and *σ*_2_ = 1). Then, a second increase in the reinfection risk, introduced by scale parameter *σ*_2_, was simulated after 31 March 2022 (*σ*_1_ = 2.8 and *σ*_2_ varied between 1 and 3). This second increase was introduced to assess the method’s ability to detect further increases in the risk of reinfection after the Omicron wave.

We measured the Gelman-Rubin convergence diagnostics for *κ*_2_,*λ*_2_ and $\lambda_{2}^{\prime }$ for the varied scale parameter combinations. We also measured the proportion of days where observed third infections were above the upper 95% of the projection interval, as well as the timing of the first cluster of five consecutive days of observed third infections that fell above the projection interval, denoted as *D*_*first*_, which can be used to detect real-time increases in the risk of third infections. In simulations with no increase in third infection risk after 31 March 2022 (i.e., when *σ*_2_ = 1), the existence of *D*_*first*_ indicates a false positive detection of an increase in the risk of third infections, and therefore we measure the specificity of the model for each value of the scale parameter *σ*_1_ as

$$specificity=1-\frac{Number\;of\;runs\;where\;D_{first}\;exists\&all\;parameters\;converged}{Number\;of\;runs\;where\;all\;parameters\;converged}$$

The model and MCMC fitting procedure were implemented in the R Statistical Programming Language [version 4.3.1 (2023-06-16)]. The code and simulated data for the generalised model is available on Github at https://github.com/SACEMA/reinfectionsBelinda.

## Results

### Data used in the third infections fitting procedure

**Fig 1:**
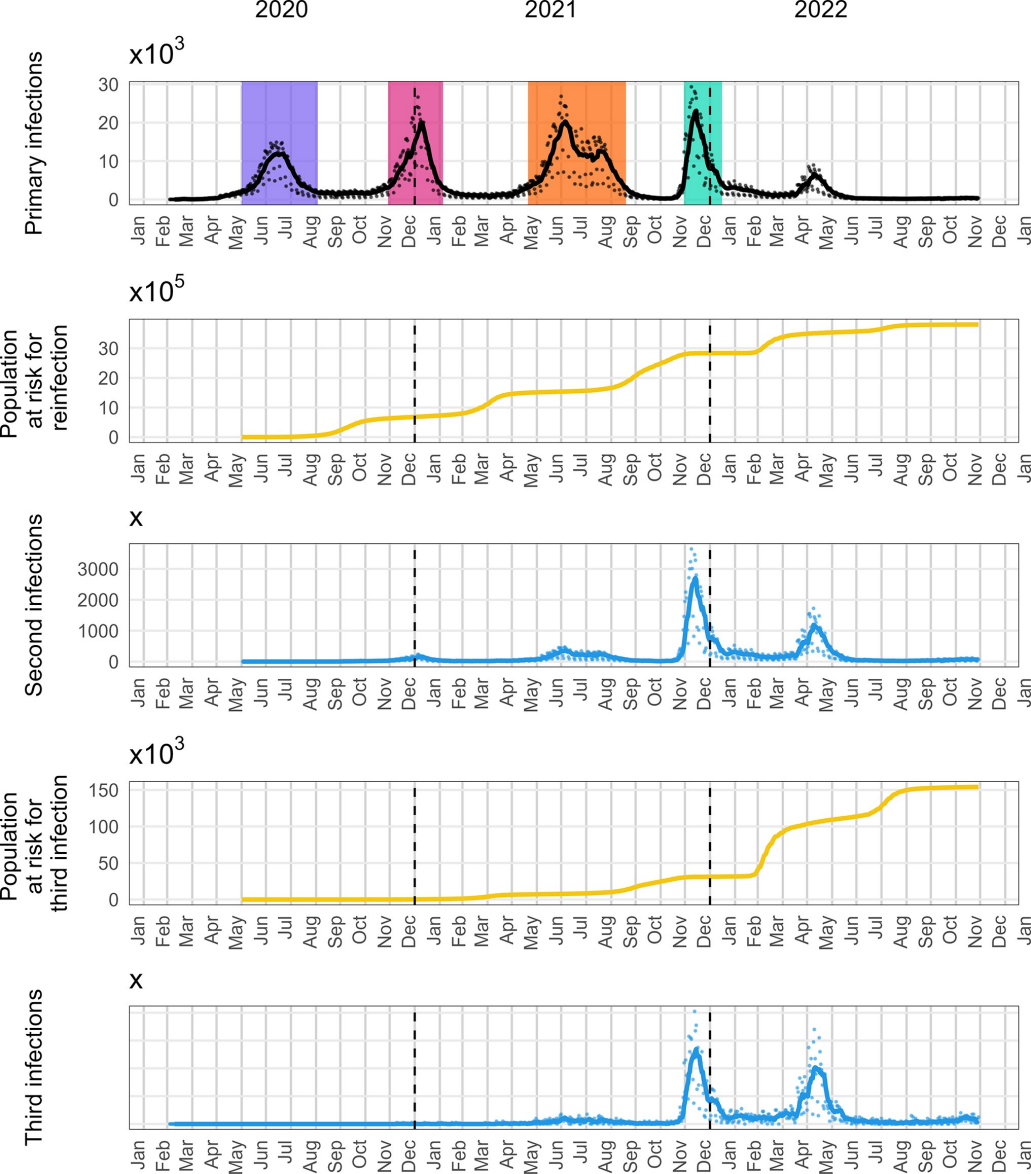
Trends in primary, second and third infections in South Africa (4 March 2020 to 29 November 2022). Panel A displays the number of observed primary infections over the period. Panel B indicates the number of individuals eligible for a second infection. Panel C presents the observed second infections. Panel D illustrates the population eligible for a third infection and Panel E depicts the observed third infections.

In Fig 1, the number of primary, second, and third infections reported in South Africa from 4 March 2020 to 29 November 2022 is depicted, along with the number of people eligible for each category.

### Model fitting

**Fig 2:**
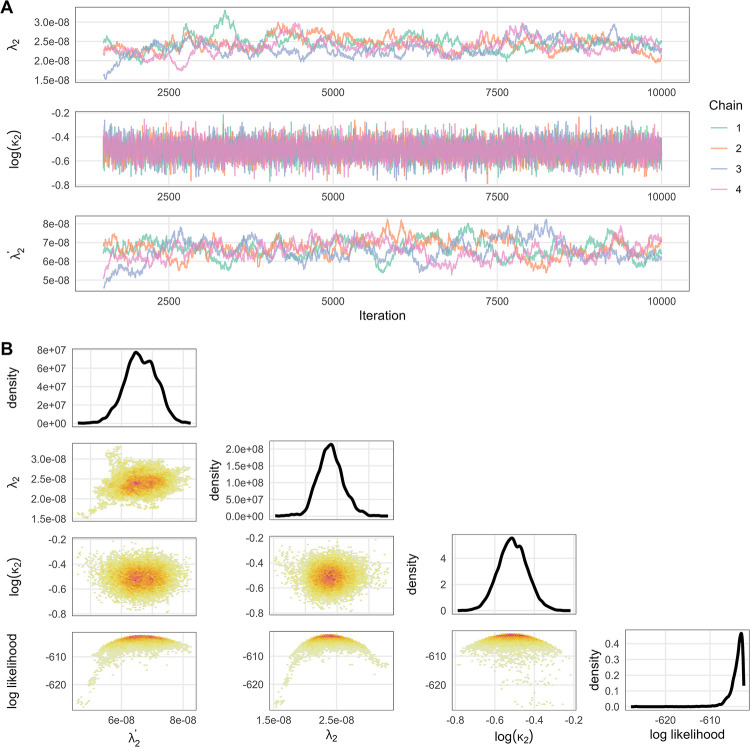
Convergence diagnostics plot when fitting $\lambda_{2},\;\lambda_{2}^{\prime }$ and **κ**_**2**_ to South African data. The top left panels show the trace plots for each parameter. On the right, the Gelman-Rubin convergence diagnostics indicate the convergence status of the parameters. The bottom panels represent density plots of the fitted parameters.

The model was fitted to South African data of reported third infections through the Omicron period up to 31 January 2022 and the parameters ($\lambda_{2},\;\lambda_{2}^{\prime }$ and *κ*_2_) converged well, with the Gelman-Rubin diagnostic values falling below 1.1. The convergence diagnostic for $\lambda_{2}^{\prime }$ was slightly higher (around 1.05) than for *λ*_2_ and *κ*_2_ (approximately 1.01 and 1.005 respectively, Fig 2).

### Model prediction

**Fig 3:**
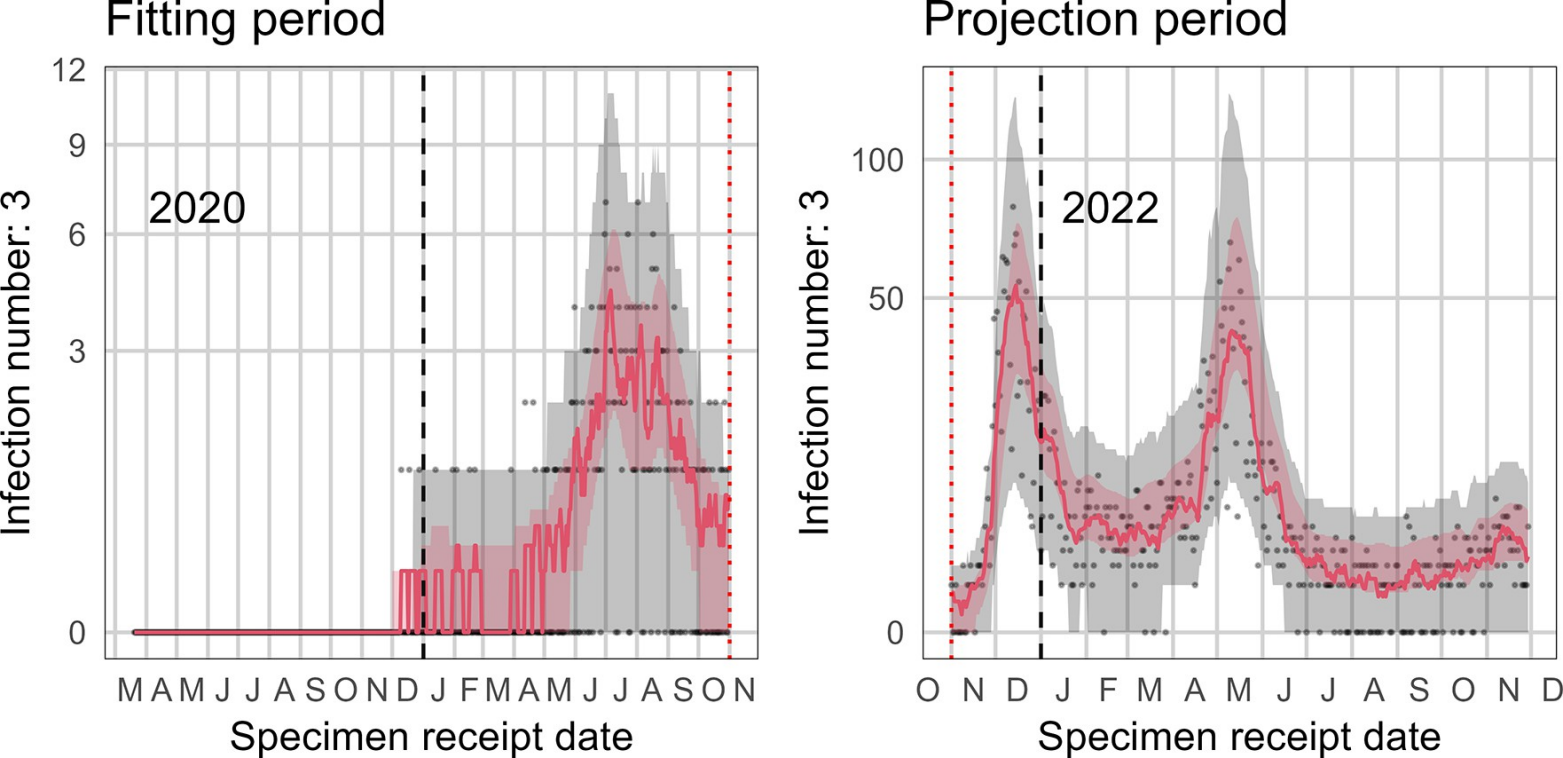
Simulation plot published in the NICD reinfections report with data fit through 31 January 2022 (https://www.nicd.ac.za/wp-content/uploads/2022/12/SARSCoV2-Reinfection-Trends-in-South-Africa_2022-12-07.pdf). The left panel represents the fitting period, and the right panel the projection period. The red band represents the 95% projection interval for the 7-day moving average of simulated third infections for that day and the grey band represents the daily simulated third infections. The red solid line depicts the 7-day moving average of observed third infections while the grey dots represent the daily numbers of observed third infections.

Fig 3 shows the 95% projection interval of expected third infections (both the 7-day moving average and the daily third infections) and the observed third infections when the model was fitted to South African data through the first Omicron wave (up to 31 January 2022), and used to project third infections. From May to November 2022, the number of observed third infections (red solid line) reaches the lower edge of the band of the 95% 7-day moving average projection interval of third infections (red band), showing a potential decreased risk of third infections. No further increase in the risk of a third infection was detected after the first Omicron wave.

### Model validation

After running the model fitting and model projection 20 times for each value of *σ*_1_ and fixing *σ*_2_ = 1, the negative binomial dispersion parameter (*κ*_2_) mostly converged when *σ*_1_>1.6. The proportion of runs where *κ*_2_ converged increased as *σ*_1_ increased, due to increased numbers of third infections. For more than 0.75 of the runs for each value of *σ*_1_ the third infection hazard coefficient before the first Omicron wave (*λ*_2_) converged, whereas the third infection hazard coefficient for after the first Omicron wave ($\lambda_{2}^{\prime }$) converged in all the runs (S3 Fig in S1 File).

The specificity (proportion of runs where an increase in the risk of third infection was not detected when there is no increase in third infection risk in the generated data, *σ*_2_ = 1) was 0.74 and higher for all values of *σ*_1_ (S2 Table in S1 File). The proportion of observed third infections above the 95% projection interval remained below 2.5%, except one run where *σ*_1_ = 3 which resulted in 5% of third infections above the projection interval.

When fixing *σ*_1_ = 2.8 and varying *σ*_2_ with values of 1.2, 1.5 and 2, the median of *D*_*first*_ from the runs where all the parameters (*κ*_2_,*λ*_2_ and $\lambda_{2}^{\prime }$) converged decreased from 26 days to 7 days as *σ*_2_ increased from *σ*_2_ = 1.2 to *σ*_2_ = 2 (S4 Fig in S1 File), and *D*_*first*_ did not exist in most cases where *σ*_2_ = 1 (specificity was 0.89). The proportion of points above the projection interval was 0.01 when *σ*_2_ = 1 and gradually increased to 0.45 when *σ*_2_ = 2 (S4 Fig in S1 File).

## Discussion

In this study, the method used to detect changes in the risk of reinfection was successfully generalised to detect the risk of multiple reinfections and validated for third infections in South Africa. The output of the method for third infections was used by the NICD in their monthly report for monitoring third infection trends [6] and will continue to contribute to surveillance efforts, particularly with the emergence of potential SARS-CoV-2 variants exhibiting immune escape. Applying the generalised model to data up to 29 November 2022 revealed no additional increase in the risk of third infection beyond the increase observed in the first Omicron wave compared to previous variants. With the extended method, we have demonstrated that we would have detected increases in the third infection risk during the fifth wave if such an increase existed.

We performed a simulation-based validation of the method, where simulated data on third infections with SARS-CoV-2 were fitted and projected. The model is robust to changes in the risk of third infections when we fitted an additional parameter that represents the second and third infection hazard coefficient during waves where the reinfection risk is higher. When the increase in the second and third infection risk in the simulated data used for the validation was low, the negative binomial dispersion parameter did not converge in some runs. This is due to an insufficient number of simulated third infections to properly inform the parameter, whereas with the higher increase in the risk of second and third infection, more data were generated to properly inform the dispersion parameter. The specificity, which assesses the method’s ability to avoid false positive detections of third infection risk increases during the projection period, was generally high for most scale values representing the initial rise in third infection risk (first Omicron wave). This suggests that the model effectively distinguished increases in the risk of reinfections from random fluctuations or noise in the data. When introducing an additional increase in the third infection risk after the additional hazard coefficient parameter is introduced), the method detects the simulated increase in the risk of third infection even for the smallest increase we investigated (*σ*_2_ = 1.2). The proportion of points above the projection interval after the introduction of the additional increase in third infection risk was only 45% when the increase was 100% (*σ*_2_ = 2), which could be due to the low number of observed third infections after the fifth wave, likely driven by reduced testing.

As we look towards applying this model to more complex scenarios, such as the risk of infections beyond the third infection, further validation is necessary. Incorporating prior knowledge and additional parameters, such as introducing a third lambda parameter to account for changing reinfection risks, will be important in ensuring accuracy. Additionally, variations in vaccine coverage across different populations may significantly influence reinfection risks and should be considered in future model applications.

While our model provides valuable insights, it is limited by its sensitivity to low counts of observed reinfections, as sufficient case counts are required for parameter convergence. Pandemic fatigue, which leads to less testing and consequently lower numbers of observed reinfections, impacts the method’s applicability in a real-life situation. With low numbers of observed multiple reinfections, the model is less likely to detect increases in the risks of multiple reinfections.

## Conclusion

The catalytic model was effectively generalised to detect increases in the risk of *n*^*th*^ infections. The method was applied to the observed third infections in South Africa to detect increases in the risk of third infection, and simulation-based validation showed its robustness in detecting increases in the risk of third infections of different magnitudes. The generalised method could contribute to future detection of increases in the risk of *n*^*th*^ infections by SARS-CoV-2 or other pathogens with similar reinfection dynamics.

## Supporting information

S1 File Supplementary materials. (PDF)
