# Supplementary material for: A generalised catalytic model to assess changes in risk for multiple reinfections with SARS-CoV-2
Source: PLoS One. 2025 Jan 2;20(1):e0315476. doi: 10.1371/journal.pone.0315476 (PMC11694959; doi:10.1371/journal.pone.0315476)
Supplement: S1 File — (PDF) [file pone.0315476.s001.pdf]

Supplementary material

Data generation of simulation-based validation

We generated a time series for the number of observed primary infections from the simulated underlying primary infections, by drawing a binomial random variable based on the observation probability, so that the number of observed primary infections on day t are represented as $\tilde{\mathrm{i}}_{\mathrm{t}} = \mathrm{Binomial}(\mathrm{i}_{\mathrm{t}}, 0.2)$, with i_t being the number of underlying primary infections on day t.

From the number of observed primary infections, the number of underlying second infections per day was calculated as:

$$r_{1,t} = hz_{1,t}.\tilde{\mathrm{i}}_t.e_{1,t}$$

where $e_{1,t}$ is the underlying number of people eligible for second infection and calculated as

$$e_{1,t} = \sum_{t=0}^{t-89}\tilde{\mathrm{i}}_t - \sum_{t=90}^{t-1} r_{1,t}.$$

The modified second infection hazard coefficient, $hz_{1,t}$, is calculated as:

$$hz_{1,t} = \Lambda\ \sigma_t$$

where Λ is the second infection hazard coefficient, fixed at 1.38866e-08, the median of the posterior distribution fitted in Pulliam *et al* (5). The scale parameter σ_t is a multiplier on the hazard coefficient to represent the increase in second and third infection risk, and is defined as

$$\sigma_t = \begin{cases} 1\ if\ \ t \le t_1 \\ \sigma_1\ if\ t > t_1\ and\ t \le t_2 \\ \sigma_1\sigma_2\ if\ t > t_2 \end{cases}$$

We used σ_1 to represent the first increase in second and third infection risk associated with the Omicron wave and introduced an additional parameter σ_2 to evaluate whether the method can detect additional simulated increases in the risk of a third infection, introduced on $t_2 = 31\ March$ 2022. Table S1 shows how σ_1 and σ_2 were varied. The first scenario represents different levels of increased risk during the Omicron wave, but no additional increased risk after t_2. In the second scenario, we fixed σ_1 at 2.8, which was the median of the ratio of the posteriors of λ_2' and λ_2 obtained from fitting the parameters to the third infection data in South Africa.

Table S1 The values of σ_1 and σ_2 considered to generate data for the simulation-based validation.

Scenario	Values of σ_1	Values of σ_2
**1**	{1, 1.2, …, 3}	{1}
**2**	{2.8}	{1, 1.2, 1.5, 2}

The number of observed second infections on day t was calculated as:

$$\tilde{r}_{1,t} = Binomial(r_{1,t}, 0.5).$$

This was further extended to calculate the number of underlying third infections on day t, $r_{2,t}$ as

$$r_{2,t} = hz_{2,t}\ \tilde{\mathrm{i}}_t\ e_{2,t}$$

where $hz_{2,t}$ is the third infection hazard coefficient and $e_{2,t}$ is the number of people eligible for a third infection on day t, calculated as:

$$e_{2,t} = \sum_{t=0}^{t-89} \tilde{r}_{1,t} - \sum_{t=90}^{t-1} r_{2,t}.$$

We calculated $hz_{2,t}$ in a similar way as $hz_{1,t}$:

$$hz_{2,t} \;=\; \Lambda_2 \, \sigma_t$$

where Λ_2 is the third infection hazard coefficient. We fixed Λ_2 at approximately $6.6 \cdot 10^{-8}$, which is obtained from the median of the posterior distribution of the third infection hazard coefficient (λ'_2) that were fitted with the observed third infections in South Africa during the Omicron wave.

The number of observed third infections was calculated as:

$$\tilde{r}_{2,t} = Binomial(r_{2,t}, 0.35).$$

In the data generation process, we varied σ_t according to the scenarios described in Table 1. For each of these values of σ_1 and σ_2, we generated a time series of second and third infections and then, after fitting λ_2, λ'_2 and κ_2, projected confidence intervals as described above. The process was repeated 20 times with different random realisations.

Supplementary figures

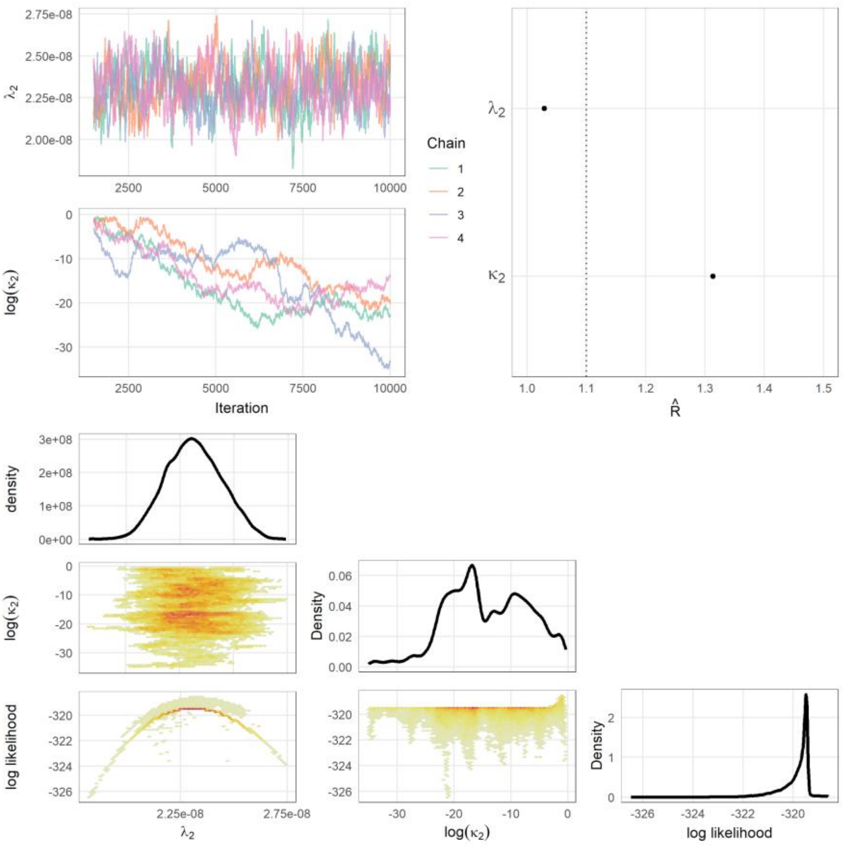

Fig S1 Convergence diagnostics with λ_2 and κ_2 fitted to the data. The top left panels show the trace plots for each parameter. On the top right is the Gelman-Rubin convergence diagnostics. The plots at the bottom are density plots of the fitted parameters. The fitting period is up until 31 October 2021 (before the Omicron period).

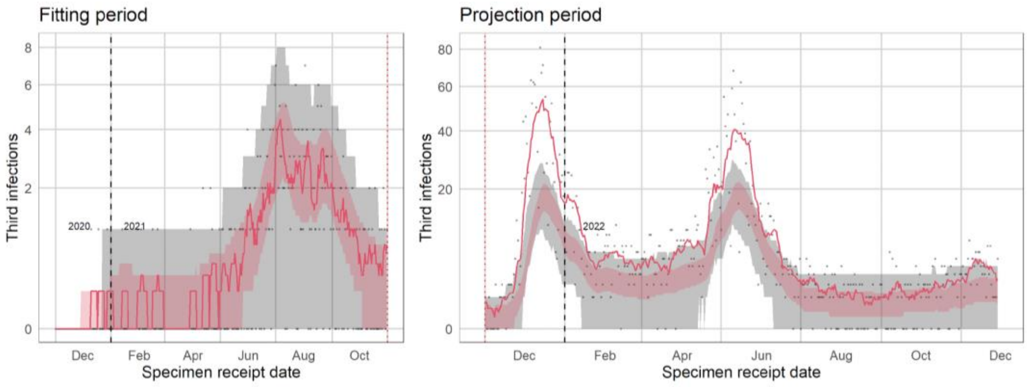

Fig S2 Simulation Plot for when only λ_2 and κ_2 were fitted up until 31 October 2021 and projected through the Omicron wave. The left side represents the fitting period, and the right-hand side the projection period. The red band is the 95% projection interval for the 7-day moving average of simulated third infections for that day and the grey band is for the daily simulated third infections. The red solid line is the 7-day moving average of observed third infections and the grey dots are the daily values of observed third infections.

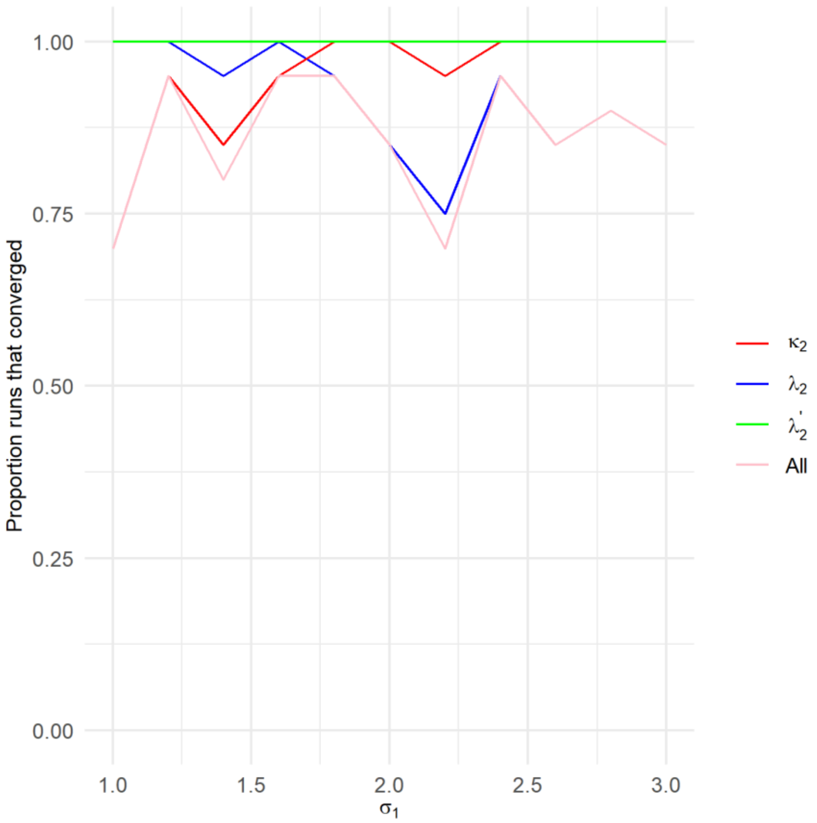

Fig S3 The proportion of the 20 runs in which κ_2, λ_2 and λ_2' converged respectively. In this plot, σ_1 is varied and σ_2 is fixed at 2.8. The pink line is the proportion of runs where all three model parameters (κ_2, λ_2 and λ_2') converged.

Table S2 Specificity measured for each σ_1 over 20 runs, after excluding runs where convergence was not achieved. Here, σ_2 is fixed at 2.8.

**Scale (σ_1)**	**Number of runs where κ_2, λ_2 and λ_2' converged**	**Specificity (conditional on convergence)**
1	14	1

1.2	19	0.95
1.4	16	0.75
1.6	19	0.74
1.8	19	0.84
2	17	0.82
2.2	14	0.86
2.4	19	0.89
2.6	17	0.82
2.8	18	0.89
3	17	0.88

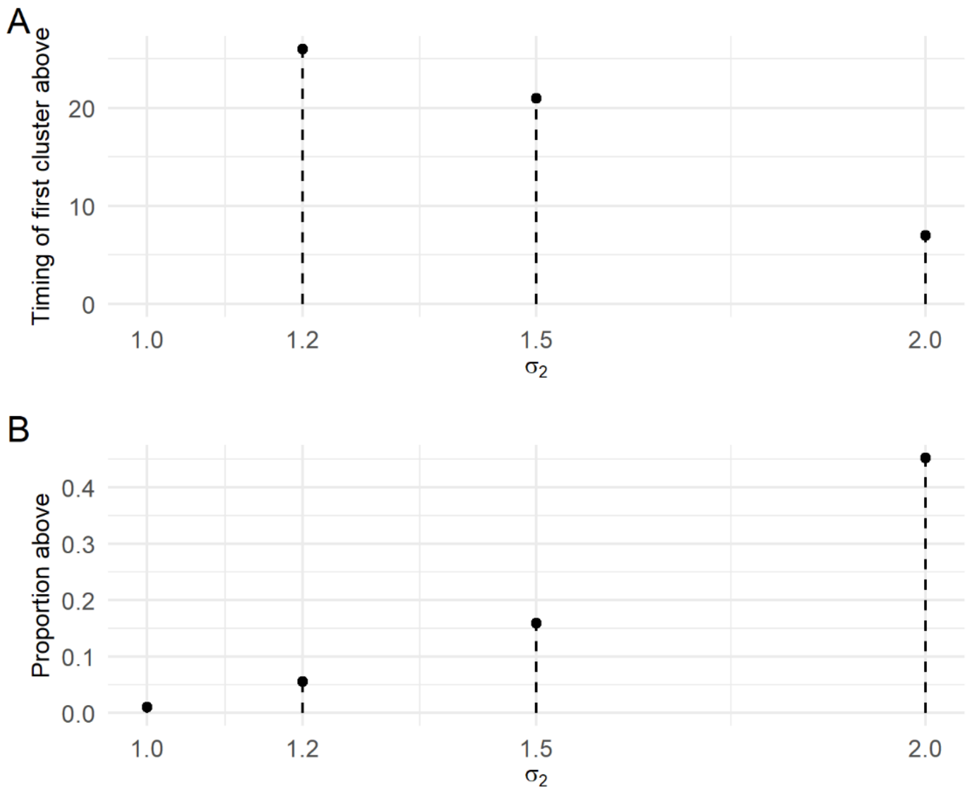

Fig S4 Results of the validation of the scenario where $\sigma_1 = 2.8$ and σ_2 varied from 1.2, 1.5 and 2. A shows the median of the timing of the first cluster of five consecutive observed reinfections, D_{first} above the projection interval for different values of σ_2. B shows the proportion points above the projection interval for different values of σ_2. Both metrics are for after the introduction of σ_2 to the data. Runs that did not converge was excluded.
